# Cloning, sequence analysis, expression of *Cyathus bulleri* laccase in *Pichia pastoris* and characterization of recombinant laccase

**DOI:** 10.1186/1472-6750-12-75

**Published:** 2012-10-23

**Authors:** Neha Garg, Nora Bieler, Tenzin Kenzom, Meenu Chhabra, Marion Ansorge-Schumacher, Saroj Mishra

**Affiliations:** 1 Department of Biochemical Engineering and Biotechnology, Indian Institute of Technology Delhi, Hauz-Khas, New Delhi 110016, India; 2 Inst.Chemistry, Department Enzyme Technology (Sekr.TC4), TU Berlin, Str. Des 17. Juni 124 D, Berlin, 10623, Germany; 3 Centre of Excellence in Biologically Inspired System Science, Indian Institute of Technology Jodhpur, Jodhpur, Rajasthan, 342011, India

**Keywords:** *Cyathus bulleri*, Heterologous laccase expression, *Pichia pastoris*, Recombinant laccase, Peptide mass fingerprinting

## Abstract

**Background:**

Laccases are blue multi-copper oxidases and catalyze the oxidation of phenolic and non-phenolic compounds. There is considerable interest in using these enzymes for dye degradation as well as for synthesis of aromatic compounds. Laccases are produced at relatively low levels and, sometimes, as isozymes in the native fungi. The investigation of properties of individual enzymes therefore becomes difficult. The goal of this study was to over-produce a previously reported laccase from *Cyathus bulleri* using the well-established expression system of *Pichia pastoris* and examine and compare the properties of the recombinant enzyme with that of the native laccase.

**Results:**

In this study, complete cDNA encoding laccase (Lac) from white rot fungus *Cyathus bulleri* was amplified by RACE-PCR, cloned and expressed in the culture supernatant of *Pichia pastoris* under the control of the alcohol oxidase (*AOX*)*1* promoter. The coding region consisted of 1,542 bp and encodes a protein of 513 amino acids with a signal peptide of 16 amino acids. The deduced amino acid sequence of the matured protein displayed high homology with laccases from *Trametes versicolor* and *Coprinus cinereus*. The sequence analysis indicated the presence of Glu 460 and Ser 113 and LEL tripeptide at the position known to influence redox potential of laccases placing this enzyme as a high redox enzyme. Addition of copper sulfate to the production medium enhanced the level of laccase by about 12-fold to a final activity of 7200 U L^-1^. The recombinant laccase (rLac) was purified by ~4-fold to a specific activity of ~85 U mg^-1^ protein. A detailed study of thermostability, chloride and solvent tolerance of the rLac indicated improvement in the first two properties when compared to the native laccase (nLac). Altered glycosylation pattern, identified by peptide mass finger printing, was proposed to contribute to altered properties of the rLac.

**Conclusion:**

Laccase of *C. bulleri* was successfully produced extra-cellularly to a high level of 7200 U L^-1^ in *P. pastoris* under the control of the *AOX1* promoter and purified by a simple three-step procedure to homogeneity. The kinetic parameters against ABTS, Guaiacol and Pyrogallol were similar with the nLac and the rLac. Tryptic finger print analysis of the nLac and the rLac indicated altered glycosylation patterns. Increased thermo-stability and salt tolerance of the rLac was attributed to this changed pattern of glycosylation.

## Background

White rot fungi have been known to completely mineralize various biopolymers such as cellulose, hemicellulose and lignin [1]. Laccase (benzenediol: oxygen oxidoreductases; EC 1.10.3.2) is one of the enzymes involved in lignin degradation. It is a phenol oxidase catalyzing four-electron reduction of molecular oxygen to water with concomitant oxidation of a phenolic substrate. This multi-copper containing enzyme has three copper centers, namely, Type 1, Type 2 and Type 3 which are distinct in terms of their spectroscopic and physical properties. The final acceptor of electrons is molecular oxygen and this binds at the Type 3 centre. The one electron oxidation in the beginning of the reaction generates a radical, which can undergo further enzyme-catalyzed oxidation or a non-enzymatic hydration or spontaneous disproportionation and/or may participate in polymerization reactions [2]. These activities can be applied in natural bioremediation processes. Due to similarity in the structure of lignin with various aromatic compounds, laccases are seen as promising enzymes for (i) dye degradation in textile waste waters (ii) wood composite production (iii) bleaching in paper and pulp industry [1,3] and, more recently, (iv) bio-catalysis [4].

The native fungi are slow growers and produce low amounts of laccase making the study and large-scale application of these enzymes difficult. Also, one organism may produce isozymes of laccase with different substrate specificities. With the objective of studying individual enzymes and achieve higher expression, a number of fungal laccase genes have been expressed in eukaryotic hosts such as *Aspergilus niger Kluyveromyces lactis*, *Pichia methanolica*, *Pichia pastoris*, *Saccharomyces cerevisiae, Trichoderma reesei* and plant (tobacco and rice) cell lines. *P. pastoris* expression system has been widely used for heterologous production of laccase from *Botrytis aclada*[5], *Ganoderma sp.En3*[6], *Fome lignosus*[7], *Pleurotus sajor-caju*[8], *Pycnoporus cinnabarinus*[9], *Pycnoporus sanguineus*[10], *Trametes* sp.AH28-2 [11], *Trametes* sp. 420 [12], *Trametes trogii*[13] and *Trametes versicolor*[14] indicating suitability of this system for laccase expression.

*Cyathus bulleri*, a member of the family Nidulariacea, has been previously reported to produce laccase with interesting biochemical properties [15]. The usefulness of this enzyme in degrading a variety of textile dyes has been shown [15-17]. The high (more than 80%) stability (> 30 days) of this laccase in a continuous membrane bioreactor [18], designed for dye decolorization, makes this a promising enzyme for large scale application. In this paper, we describe the isolation and characterization of the full length cDNA encoding this laccase. The laccase was expressed in *P. pastoris* under the control of alcohol oxidase (*AOX*)*1* promoter. The purified recombinant protein (rLac) was biochemically characterized and properties relevant to applications compared with laccase (nLac) of the native fungus.

## Results

### Sequence analysis of cloned laccase

**Figure 1:**
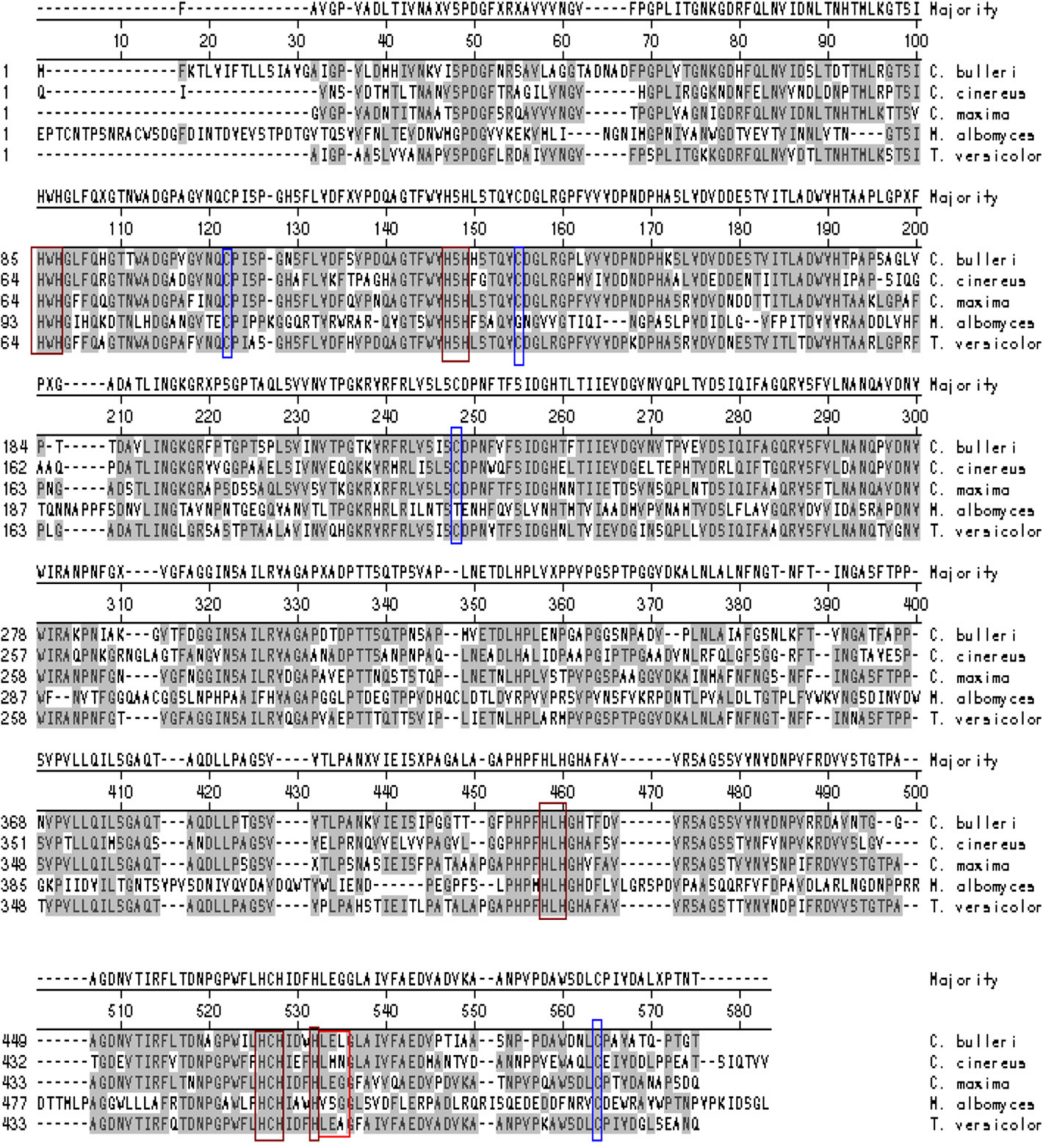
**Amino acid sequence alignment of laccase from *Cyathus bulleri *with other fungal laccases. ***Cerrena maxima *(PDB accession code 2H5U_A)*, Coprinus cinereus *(PDB accession code 1A65_A)*, Melanocarpus albomyces *(PDB accession code 2Q90_A)*, Trametes versicolor *(PDB accession code 1GYC_A). The blue boxes represent the cysteine residues present in disulphide bridges. The red boxes represent conserved copper binding domains.

Based on the 435 bp sequence obtained [19] using primers raised against the internal peptide sequence of the nLac, RACE PCR strategy was used to isolate the complete cDNA of laccase. For this, 24 h post-induction culture of *C. bulleri* was used for preparation of RNA. The length of the coding sequence was 1,542 bp and the gene encoding this protein was named as *lcc*. The von Heijne [20] signal sequence prediction was used to predict the start of the mature laccase. The predicted N-terminus matched with the reported N-terminal sequence [21] of the matured protein. Hydrophobic residues were found to be present in the central region of the putative signal peptide. No sequence similarity was observed in this pre-pro region with signal sequences of other laccases. The mature laccase polypeptide was predicted to be 497 amino acids long with a secretion peptide of 16 amino acids. The molecular weight, calculated on the basis of average isotopic masses of the amino acids, was 53,029 Da and the isoelectric point was 4.9. The laccase contained four putative N-glycosylation sites (Asn-X-Ser/Thr), at positions 37, 209, 247 and 452. Two of the sites, at positions 209 and 247, seem less likely to be glycosylated because of proline at C-terminal side of threonine [22]. Multiple sequence alignment (Figure 1) with known laccases indicated high sequence identity with basidiomycete laccases as compared to the ascomycete laccases. Highest sequence similarity (about 60%) was observed with laccases from *T. versicolor*[23] and *Coprinus cinereus*[24]. All the expected Cu(II) ligands in laccases were strongly conserved: eight histidine residues in the highly conserved motif of four His-X-His repeats that coordinate the trinuclear Type 2/Type 3 copper (shown as red boxes); additional four cysteines and histidine were also found to be strongly conserved (blue boxes) and are likely to be important in binding to Type 1 copper site.

### Expression of cloned laccase in *P. pastoris*

**Figure 2:**
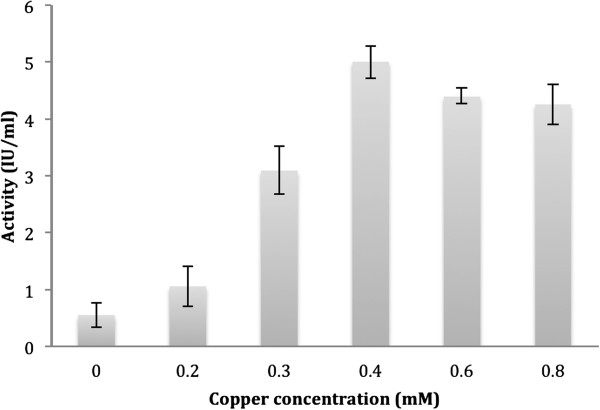
**Effect of addition of copper salt in induction medium. **Extracellular level of laccase in *P. pastoris *clone pPICZαB *lcc-*5 after addition of different concentrations of copper sulfate at the beginning of the induction phase.

**Figure 3:**
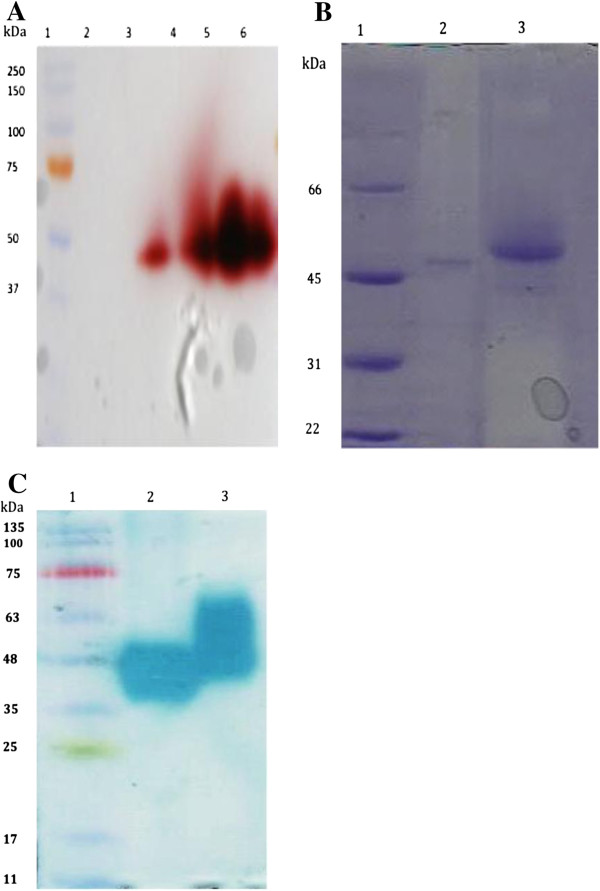
**Expression, purification and zymogram analysis of purified laccase. **(**A**) PAGE separation of proteins from culture supernatant of *P. pastoris *pPICZαB *lcc-*5 and confirmation of laccase secretion by zymogram analysis using guaiacol as substrate. Lane 1, Pre-stained molecular weight markers. Lanes 3,4: 10, 20 μl respectively of the culture filtrate. Lane 5, 20 μl of the culture filtrate in loading buffer not containing SDS. Lane 6: 20 μl of the culture filtrate in loading buffer not containing β–mercaptoethanol. (**B**) SDS-PAGE analysis of the purified laccase. Lane 1, molecular weight markers. Lane 2, purified nLac (~5 μg). Lane 3, purified rLac from *P. pastoris* (~7 μg). (**C**) PAGE-Zymogram analysis using ABTS as a substrate. Lane 1, Pre-stained molecular weight markers. Lane 2, purified nLac. Lane 3, purified rLac.

The *lcc* gene was inserted into the *P. pastoris* expression vector pPICZα in frame with the α–factor secretion signal gene, under the control of the *AOX1* promoter. The construct was introduced into the yeast genome and extracellular expression of laccase was confirmed (under methanol inducible conditions) by plate assay on 2,2^′^-Azino-bis (3-ethylbenzothiazoline-6-sulfonic acid) or ABTS. A number of clones displayed green color on plates and this indicated correct processing of the signal sequence. All the laccase producing transformants were found to be Mut^+^ (methanol utilization phenotype). The clone pPICZα *lcc-*5, which showed deepest green color on the plates, was chosen for expression studies in liquid medium (Invitrogen Basal medium). Maximum laccase activity of 600 U-720 U L^-1^ was observed 3 days after initiation of induction by 1.0% methanol. Effect of addition of copper sulfate at different times, post induction, was investigated and maximum laccase activity of ~7200 U L^-1^ was obtained at salt concentration of 0.4 mM (Figure 2). This represented an increase of about 12-fold over control cultures where no copper was added. The PAGE-zymogram analysis of the concentrated culture filtrate, carried out on guaiacol, confirmed the expression of laccase in active form (Figure 3A, lanes 3,4). For equal volume of concentrated culture filtrate loaded on the gel, omission of SDS and β–mercaptoethanol in the loading buffer resulted in higher activity as judged on the gel (Figure 3A, lanes 5,6 respectively).

### Purification and characterization of rLac

Recombinant laccase expressed in *P. pastoris* was purified using ammonium sulfate precipitation, followed by chromatography on Sephadex G75 and Superdex G200 columns kept in tandem. A summary of the purification steps is shown in Table 1. A final specific activity of 85 U mg^-1^ was achieved representing a purification fold of ~4. A total enzyme yield of 24% was obtained. A single protein band was detected on SDS-PAGE indicating electrophoretic homogeneity of the sample (Figure 3B, lane 3) and a relative molecular mass of ~60 kDa was estimated. The mass of this purified rLac was slightly higher than the nLac (Figure 3B, lane 2) which has been reported to be of ~58 kDa [15]. The laccase activity of the purified band was confirmed by zymogram analysis using ABTS as a substrate, the oxidized radical of which was visualized as a green colored band (Figure 3C, lanes 2 and 3). The gel showed diffused band with both nLac and rLac and most importantly, the higher molecular weight of the *Pichia* expressed laccase was observed more clearly in the zymogram analysis (Figure 3C, Lane 3). The spread of the rLac was more heterogeneous compared to the nLac. The same observations were made when the proteins were stained with a dye specific for glyco-proteins. The *Pichia* expressed rLac moved at a higher position indicating higher molecular mass of this laccase (Additional file 1: Figure S1). The pH and temperature optimum of the rLac were measured using ABTS as the substrate and found to be 4.0 and 55°C respectively. For rLac, stability increased from pH 2 to 7 (where it was most stable). The temperature stability was studied and half-life values were determined to be 38.5 h (at 25°C), 25.7 h (at 30°C), 8.1 h (at 35°C), 7.6 h (at 40°C), 2.3 h (at 50°C) and 0.6 h (at 60°C). These were much higher than the values reported earlier [15] for the nLac which were 63 min (at 35°C), 48 min (at 40°C), 18 min (at 50°C) and 4 min (at 60°C). When stored at 4°C, rLac was as stable as the nLac. The kinetic parameters were determined for the rLac on ABTS, guaiacol and pyrogallol and compared with the values obtained with the nLac. Similar values for Km and Vmax were obtained (Additional file 2: Table S1) indicating functional similarity of the *Pichia* produced enzyme with that of the native fungus.

**Table 1:** **Summary of purification of laccase secreted by *P. pastoris *pPICZαB *lcc*-5**

**Purification Steps**	**Total activity (U)**	**Total protein (mg)**	**Specific activity (U/mg)**	**Yield (%)**	**Purification fold**
Crude extract	370	18	20.5	100	1
Ammonium sulfate precipitation	138.24	3.6	38.4	37.3	~1.9
Sephadex G75 + SuperdexG200	90	1.06	84.9	24.3	~4.0

**Figure 4:**
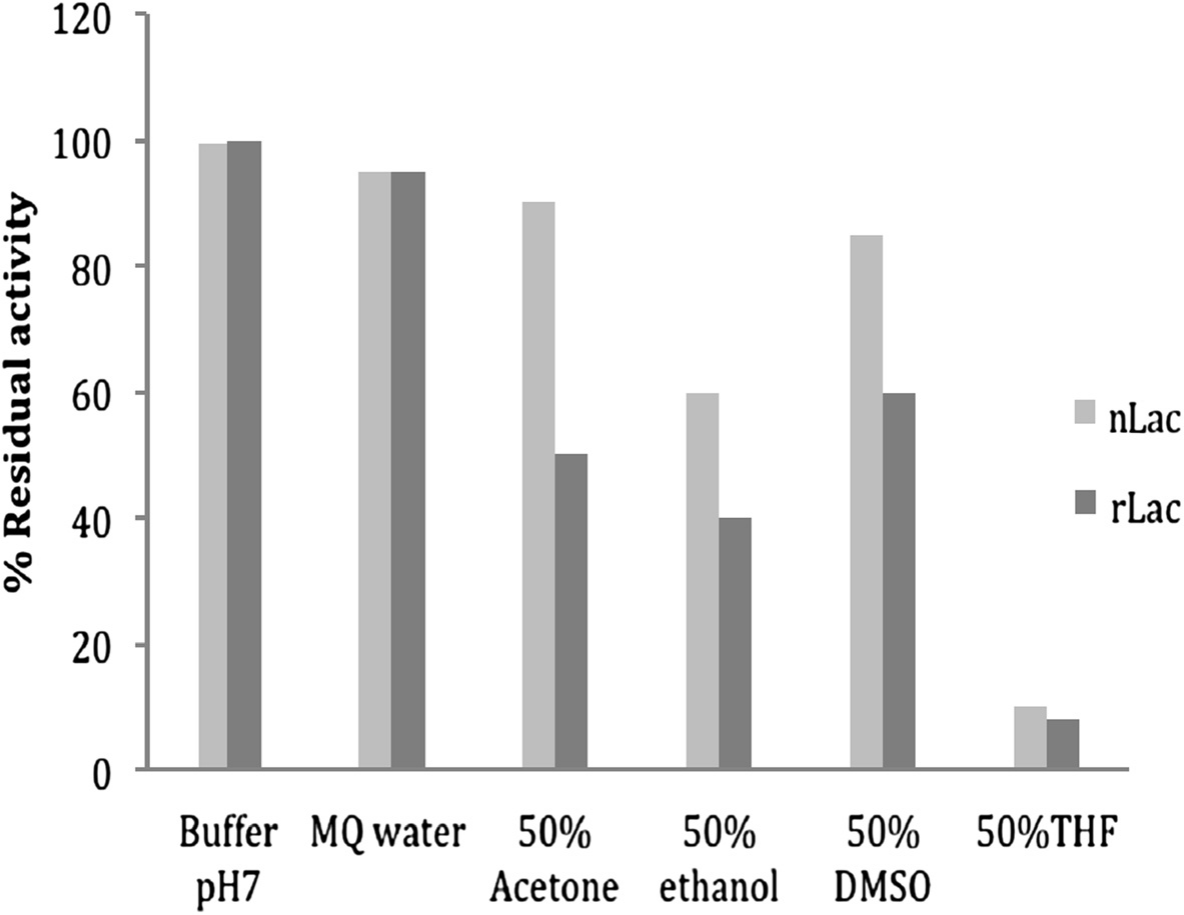
**Residual activity of laccase in the presence of organic solvents. **Percent residual laccase activity after incubation of purified enzyme for 3 h in the presence of various solvents as indicated on X axis. Hundred % activity corresponds to 0.5 U in the total reaction mixture. Light grey: nLac, Dark grey: rLac.

**Figure 5:**
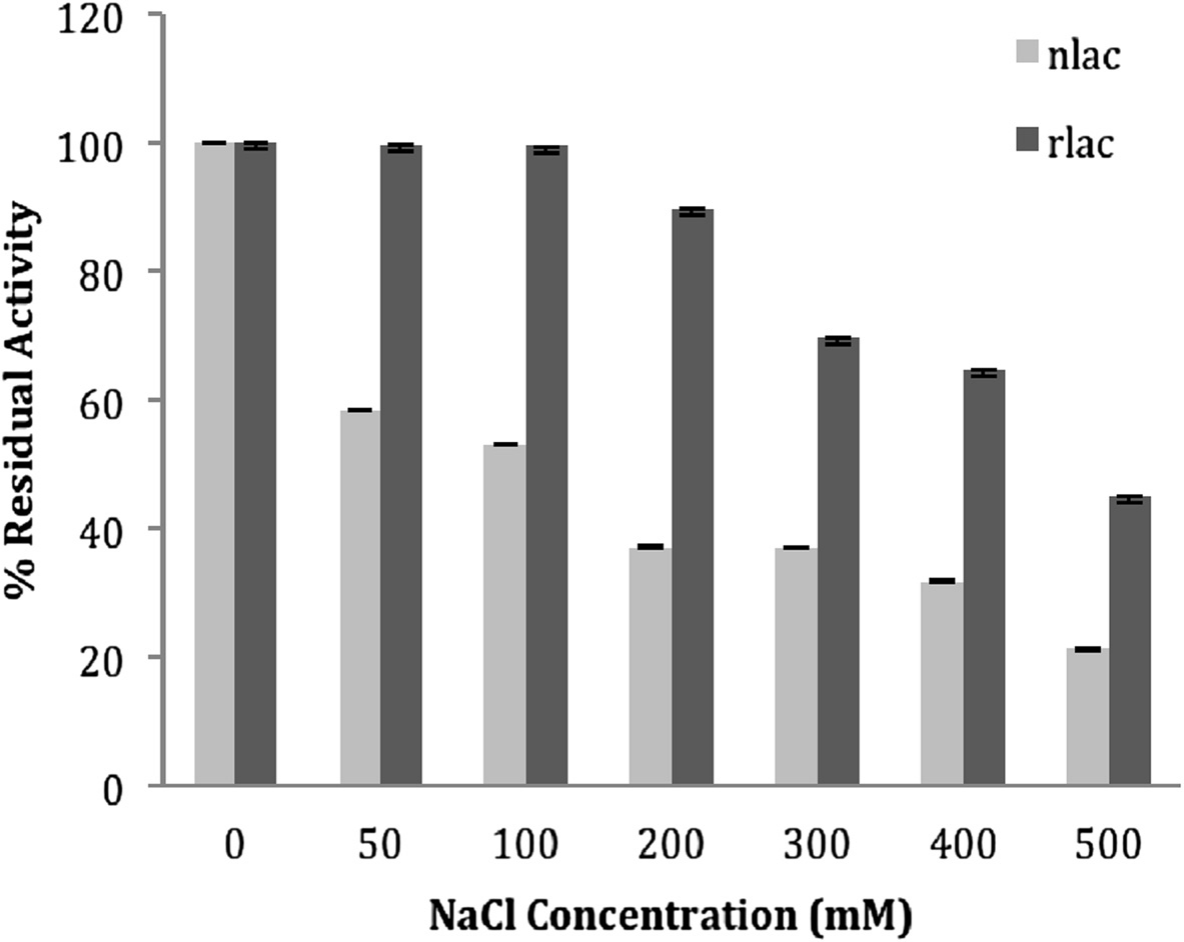
**Residual activity of laccase in the presence of NaCl concentration. **Percent residual activity after incubation of purified enzyme for 2 h in the presence of different NaCl concentrations. Hundred % activity corresponds to ~1.0 U laccase activity in the total reaction volume. Light grey: nLac, Dark grey: rLac.

The effect of various water miscible organic solvents (acetone, ethanol, dimethylsulfoxide or DMSO) and sparingly soluble solvents (tetrahydrofuran or THF, methyl tertiary butyl ether or MTBE), commonly used for synthesis of aromatics, was investigated on laccase activity and the results are shown in Figure 4. Both rLac and the nLac were stable in these solvents (except for THF) up to 3h at 4% (v/v) concentration. However, at higher solvent concentration of 50% (v/v), differences were noted and nLac retained between 60-90% activity in water miscible solvents. The rLac was slightly less stable (40-60%). In sparingly soluble solvents, both the native and the recombinant enzyme were inactivated by 90%. Maximum inactivation of activity (wherever applicable) occurred during either the first hour of incubation (with acetone and ethanol) or during the first two hours (DMSO) after which the rates were stabilized. At 70% solvent level, both rLac and the nLac were inactivated. With chloride ions, the stability was monitored for 2 h and the data is shown in Figure 5. Recombinant Lac was found to be more stable at all concentrations of chloride compared to the nLac. In the high concentration range of 300–500 mM, more than 50% residual activity was observed.

### MALDI-TOF MS and peptide mass fingerprint analysis of rLac and nLac

**Figure 6:**
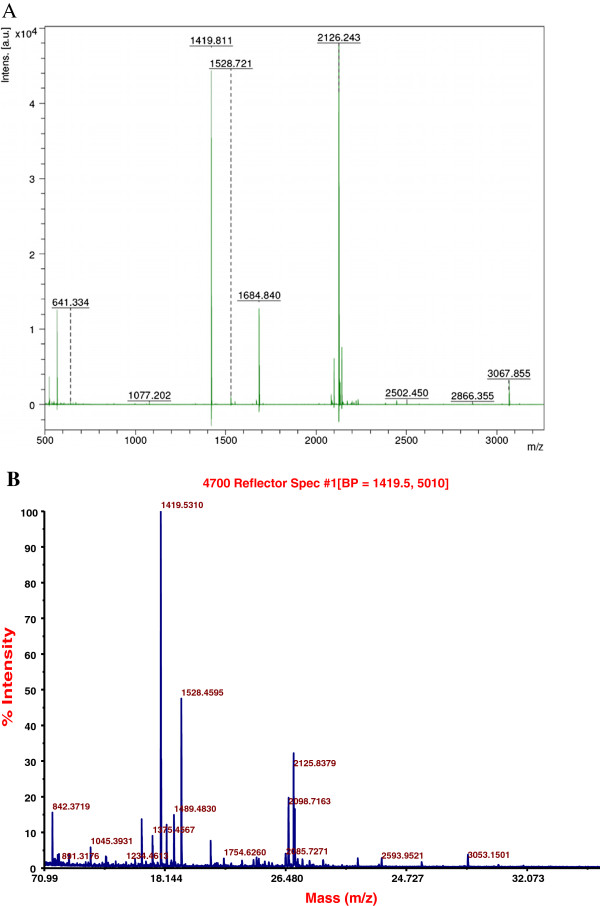
**MALDI-TOF mass spectra of peptide fragments of laccase. **Mass fingerprint analysis of peptides arising from SDS-PAGE separated and *in situ *trypsin digested purified nLac (**A**) and rLac (**B**).

For identification of the rLac expressed in *P. pastoris*, tryptic *in-gel* digestion of the purified commassie stained band (Figure 3B, lane 3) was performed. Masses of the tryptic fragments, determined by MALDI-TOF MS analysis, were compared to the masses obtained with the nLac and the spectra are shown in Figure 6A (nLac) and Figure 6B (rLac). The details of various peaks are provided in Additional file 3: Table S2 and Additional file 4: Table S3 respectively. Since glycosylation affects only a few peptides, the theoretical peptides expected of this laccase on complete digestion with trypsin can be predicted and are given in Additional file 5: Table S4. Four of the peptides in the rLac at m/z values of 1419.53, 1528.45, 2098.71, and 2125.83 (Figure 6B) were identified and matched exactly with the peptides of m/z 1419.81, 1528.72 + 1684.84 (with an additional D), 2099.10 (see Additional file 4: Table S3) and 2126.24 obtained from the nLac (Table 2). This confirmed that rLac was the same as the purified nLac. For rLac and the nLac, additional peptide fragments with m/z values of 2593.95 (Figure 6B) and 2594.041, 2594.049 (Additional file 4: Table S3) and 2132.1 (Additional file 3: Table S2), 2866.35 (Figure 6A) were detected in addition to several un-identified peptides. Glycomod tools [25] were used for calculating the theoretical mass of the potential glycosylated tryptic fragments (only 2 out of the 4 possible sites due to the presence of Pro at the carboxy-terminus) and their possible structures. Based on this, the peak observed at 2593.95 was identified to be of the peptide 442 DAVNTGGAGDNVTIR 456 with the assigned glycan structure of (Hex)_4_ (HexNac)_2_ (Sulph/Phos)_1_ . For the nLac, the tryptic fragment at m/z of 2132.1 was concluded to be 442 DAVNTGGAGDNVTIR 456 with the glycan structure of (Hex)_1_(HexNAc)_2_(NeuGc)_1_. The details are provided in Table 2.

**Table 2:** Summary of major peptide fragments obtained from trypsin digestion of (A) nLac and (B) rLac with % Relative intensity, sequence and assigned glycan structures

**A**			
**m/z**	**Relative Intensity**	**Peptide sequence**	**Assigned glycan structure**
1419.811	100	GVTFDGGINSAILR	
2126.243	87.55	GRFPTGPTSPLSVI**NVT**PGTK	
1684.84	28.65	SAGSSVYNYDNPVRR.D	
3067.855	4.00	VIEISIPGGTTGFPHPFHLHGHTFDVVR.S	
2132.145	19.01	DAVNTGGAGD**NVT**IR	**(Hex)**_**1 **_**(HexNAc)**_**1 **_**(NeuGc)**_**1**_
**B**			
1419.531	96.35	GVTFDGGINSAILR	
1528.459	45.54	SAGSSVYNYDNPVR	
2125.837	24.34	GRFPTGPTSPLSVI**NVT**PGTK	
2098.716	16.27	YSFVLNANQPVDNYWIR	
	12	GPLVVYDPNDPHK	
2593.952	1.61	DAVNTGGAGD**NVT**IR	**(Hex)**_**4 **_**(HexNAc)**_**2 **_**(Sulph)**_**1**_
			**(Hex)**_**4 **_**(HexNAc)**_**2 **_**(Phos)**_**1**_

## Discussion

In the last few years laccases have been identified as important enzymes for application in the environment sector as well as for production of high value aromatics [4,26]. Almost all laccases are produced at low levels in the native fungi. Multiple isozymes of laccase have also been reported in numerous fungi making the study of individual enzymes difficult. Cloning and expression of genes provides an opportunity to overproduce and study these enzymes individually. Based on our previous studies [15,18], wherein a laccase was purified from *C. bulleri* and investigated for its application in degradation of textile dyes, the cDNA encoding this enzyme was isolated in the present study and expressed in *P. pastoris.* Although many white-rot fungi exhibit multiple isozymes of laccase, we and others [21] have only observed one laccase in *C. bulleri*. To isolate the full length cDNA encoding this laccase, RLM-RACE technique was used. This method has been found to be very useful in isolating gene sequences using primers from the known and the conserved regions [27] of the genes. Primers were designed in the present study, based on the previously sequenced 435 bp sequence [19], and used to obtain the complete coding sequence through the primer walking technique. A comparison of the complete protein sequence with other laccases indicated high similarity to basidiomycete laccases, especially in the copper binding regions, with all His and Cys residues conserved. The internal peptide sequences reported [15] for the nLac were identified in the rLac indicating that the cloned gene was that of the enzyme studied earlier. The reported [21] N-terminus sequence of the laccase was also identified in the deduced amino acid sequence. It has been proposed [28] that for Type 1 copper ligand, residues located 10 amino acids downstream of the conserved Cys affect the redox potential and this provides a basis for classification of laccase under class 1 (Met), class 2 (Leu) or class 3(Phe). The sequence LEA adjacent to the last conserved His is conserved in laccases of high redox potential with Ala at the most being replaced by other residues. This contrasts with the laccases of low redox potential which have a sequence of VSG replacing the LEA tripeptide. The presence of Leu at the Type 1 copper binding position and LEL tripeptide (Figure 1, last row, red box #2) suggests the *C. bulleri* laccase to be a high redox potential enzyme. Moreover, the presence of Glu 460 and Ser 113 at the equivalent conserved positions further strengthens this hypothesis. The sequence also showed the presence of pre-sequence in agreement with its extracellular localization. The sequence conservation in this region between various fungi was observed to be poor indicating the use of different secretion peptides in different fungal species.

The *C. bulleri* laccase was expressed at a level of 600–720 U L^-1^ using pPICZαB yeast shuttle vector*.* In general, low heterologous expression of fungal laccase has been reported in *P. pastoris* (less than 1000 U L^-1^) compared to the expression levels in the native fungi. Enhancement in extracellular laccase activity has been reported by addition of copper sulfate [7,9] to the culture medium of *P. pastoris*. Mutagenesis of the structural gene followed by expression in *P. pastoris* also lead to enhanced expression of *Trametes* sp. AH28 -2 laccase [12]. In the present work, optimization of the time of addition and concentration of copper salt lead to an increase in laccase activity to ~7200 U L^-1^ in 6 days. While this effect of copper has been reported at the level of transcription, mediated by copper-dependent responsive element in some fungi [29], no such effect is expected in the recombinant *P. pastoris* as the laccase gene is transcribed under the control of the *AOX1* promoter. Addition of copper salts in the medium does not affect extracellular laccase activity in the native *C. bulleri* either (unpublished data). Since laccases are metallo-proteins, it is likely that the addition of copper allows the excreted laccase in *P. pastoris* to fold correctly in the culture filtrate.

The rLac appeared as a 60 ± 5 kDa protein, slightly higher and more heterogeneous when compared to the nLac (58 ± 5 kDa) [15] and these differences were attributed to increased glycosylation of the rLac in *P. pastoris* (see below). While the kinetic parameters against ABTS, guaiacol and pyrogallol were found to be similar (Additional file 2: Table S1) for the native and the rLac, the latter was found to be more thermo-stable. This thermo-stability is likely to be on account of higher glycosylation in *P. pastoris*. It has been proposed that the glycans, being highly hydrophilic in nature, contribute to the stability by associating covalently to the amino acid residues present on the surface of the protein molecules [30]. The higher stability is expressed by higher melting temperature. The recent work on the engineered SH3 domain variants also clearly suggested that glycosylation can enrich as well as modulate the biophysical properties of proteins and could, in fact, be used as an alternative way to design thermally stabilized proteins [30]. In this study, we can also correlate the tolerance to organic solvents as a by-product of this altered glycosylation pattern. As observed, the rLac produced in *P. pastoris* exhibited higher tolerance towards various water-miscible organic solvents compared to the native laccases from *T. versicolor* and *Pleurotus ostreatus*[31]. Between 40-60% residual activity was observed at 50% (v/v) in all these solvents after 3 h of incubation. While these values were slightly lower than those observed for the nLac (Figure 4), these are still high and useful for its use in organic synthesis work. Interestingly, both the rLac and the nLac were equally unstable in THF (solvent of a higher log P value)which is likely to have distorted the enzyme hydration and distort the conformation leading to a drastic decrease in enzyme activity. It has been observed that laccase structure, stability and activity are affected by water miscible solvents through direct interaction with enzyme and through its affect on water activity (a_w_) [32]. Although Farnet et al. [33] have observed a high IC_50_ values (30-60%) of the *Marasmius quercophilus* laccase in different solvents but the enzyme was not incubated for longer time periods and thus their data cannot be compared to our results.

Laccases are generally inhibited by chloride ions, an important component in dye wastewaters, which limits its use in treatment plants. Chloride ions directly affect the conversion process through their intrinsic effects on rate constants mediated through availability of Type 2 and Type 3 copper atoms in the active site [34]. Higher resistance to chloride ions (after 2 h incubation) was observed for the rLac of *C. bulleri* (Figure 5) when compared to the nLac. A chloride tolerant laccase having IC_50_ of 1.5 M was recently reported [5] but again, the enzyme was not incubated for long time periods and hence cannot be compared to the laccase expressed in this study.

For many of the differences observed between the rLac and the nLac, a detailed comparison of the trypsin digested peptide fragments was made. Several peptides were found to be identical confirming the expression of the same laccase in *P. pastoris*, as reported previously from our group. Out of the 4 putative glycosylation sites, only 2 were likely to get glycosylated [20]. Differences in the glycosylation patterns, leading to generation of a spectrum of different peptides, were observed. Software tools were used to identify these and the fragment with m/z of 2593.9521 (obtained from the rLac) was concluded to represent the aa sequence 442–456 with possible glycan structure of (Hex)_4_ (HexNAc)_2_ (Sulph/Phos)_1_. The corresponding fragment from the nLac was identified at 2132.1 m/z with an assigned structure of (Hex)_1_(HexNAc)_2_(NeuGc)_1_. While theoretically, additional peptide (28 VISPDGFNRSAVLAGGTADNADFPGPLVTGNK 38) is predicted to undergo glycosylation and may indeed do so, this is not likely to be detected by MALDI-TOF MS, as the size of this exceeds the detection limits of the system.

## Conclusion

The full length cDNA sequence of *C. bulleri* laccase is reported in this paper. The gene was efficiently expressed under the control of the *AOX1* promoter and secreted in the culture supernatant of *P. pastoris* . Sequence analysis indicated this to code for a high redox laccase. Optimization of the time of addition and concentration of copper salts resulted in laccase activity of ~7200 U L^-1^. The laccase was purified to homogeneity and found to be of a higher mol wt compared to the nLac. An investigation of biochemical properties of the rLac indicated it to possess higher thermo-stability and tolerance towards chloride ions compared to the nLac. A comparison of the peptide mass fingerprint data with the nLac indicated presence of fragments, not observed in the nLac, which were attributed to different pattern of glycosylation in the *P. pastoris* and which are likely to have contributed to the observed differences in some biochemical properties. The data indicate usefulness of the rLac over the nLac in specific areas of applications.

## Methods

### Organisms, plasmids and enzymes

*C. bulleri* (Brodie) 195062 (common name: birds’ nest fungus) was from Canadian Type Culture Collection. The fungus was cultivated as described previously [15]. The *Pichia* vector pPICZαB and host *P. pastoris* X33 strain were from Invitrogen. The yeast was maintained on YPD (1% yeast extract, 2% bacto-peptone, 2% glucose). *Escherichia coli* DH5α was from Technical University, Aachen, Germany and TOP 10 cells were provided in the TOPO TA cloning kit for sequencing (Invitrogen). *E. coli* was grown in Luria-Bertani medium. Unless otherwise stated, the enzymes used to manipulate DNA or RNA were obtained from New England Biolabs, Promega or Fermentas.

### Oligonucleotides

The oligonucleotides (Sigma-Aldrich) used in the study are shown in Table 3.

**Table 3:** List of oligonucleotides used in the study

**Oligonucleotide**	**Sequence**
GeneRacer 5^′ ^Primer	5^′^- CGA CTG GAG CAC GAG GAC ACT GA -3^′^
GeneRacer 3^′ ^Primer	5^′^- GCT GTC AAC GAT ACG CTA CGT AAC G -5^′^
GeneRacer Oligo dT Primer	5^′^- GCT GTC AAC GAT ACG CTA CGT AAC GGC ATG ACA GTG (T)_24- _3^′^
GeneRacer RNA Oligo sequence	5^′^- CGA CUG GAG CAC GAG GAC ACU GAC AUG
For-gsp	GAC UGA AGGAGU AGA AA -3^′^
Rev-gsp	5^′^- TAG CGC CGG AAG CAG CGT GTA CAA CTA -3^′^
Oligo 2	5^′^- GGT GTC TGG TGC ACC GGC ATA TC -3^′^
Oligo 3	5^′^- ATT TCC CCG CGG TCA GGT GCC GGT TGG- 3^′^
	5^′^- CCG CTG CAG CCA TTG GCC CAG TTT CGGA -3^′^

### RNA isolation

*C. bulleri* cultures were grown in basal liquid medium [35] and induced with 2,6-dimethylaniline [15]. Fungal mycelium was collected 24 h after induction by filtration, washed twice with sterile phosphate buffer (20 mM, pH 7.0) and frozen in liquid nitrogen. Crushed frozen mycelium (100 mg) was used to isolate total RNA using RNeasy Plant Mini Kit (Qiagen). The quality of RNA was checked by running on agarose gel.

### Determination of laccase nucleotide sequence by RNA ligase mediated RACE-PCR

GeneRacer™ RLM-RACE kit (Invitrogen) was used to obtain 5^′^ and 3^′^ ends of laccase cDNA. Two μg of the total RNA was treated with calf intestinal phosphatase to remove phosphates from truncated and non-mRNA. The dephosphorylated RNA was given tobacco acid pyrophosphatase treatment to remove mRNA cap structure. Oligo (provided in the kit) ligated RNA was primed with oligo dT primer (Gene Racer Oligo dT Primer) and reverse transcription was carried out using Superscript II RT (Invitrogen). GeneRacer 5^′^ Primer, complimentary to the anchor sequence and Rev-Gsp primer designed from the 435 bp laccase sequence described previously [19] were used to amplify the 5^′^end. Gene Racer 3^′^ Primer, from the anchor attached to Oligo dT and For-Gsp designed from the 435 bp laccase sequence described previously were used to obtain the 3^′^end. The PCR product was sequenced (MWG DNA Sequencing Service, Germany) and complete laccase cDNA sequence deduced using primer walking technique. Sequences were aligned using Clustal V program.

### Cloning and expression of laccase gene through yeast shuttle vector

Total RNA was reverse transcribed using Oligo dT primer with M-MuLV reverse transcriptase (New England Biolabs). The von Heijne signal sequence prediction [20] was used to predict the start of the mature laccase. A 1,491 bp fragment corresponding to the laccase cDNA (without the signal peptide encoding fragment) was amplified using downstream Oligo 2 and upstream Oligo 3 generating *Sac* II and *Pst* I sites respectively. The PCR product was cloned into pCR4-TOPO vector in *E. coli* TOP 10 cells as per instructions (Invitrogen). The presence of the desired PCR product was verified by restriction enzyme digestion, agarose gel electrophoresis and sequencing. The recombinant plasmid was linearized using *Sac*I. The Easy select *Pichia* expression kit (Invitrogen) was used for heterologous expression of the laccase cDNA without its own signal peptide. The medium recipe, transformation and analysis of the recombinants were carried out as per the kit manual. *P. pastoris* X33 was transformed with *Sac* I linearized recombinant pPICZαB vector and the transformants were selected for Zeocin resistance on YPD medium. Twenty or so transformants were screened on minimal methanol plates supplemented with 0.2 mM ABTS for development of green color. One (pPIC *lcc*-5) of the recombinants (selected on the basis of development of intense green color in plate assay) was cultivated in 50 mL YPD medium in 300 ml baffled flasks. At culture OD_600_ of 2–6, the cells were harvested by centrifugation and re-suspended in buffered complex methanol medium at an OD_600_ of 1.0. The culture was monitored for 6 days for production of extracellular laccase with the induction of the promoter being maintained by daily addition of 1% (v/v) methanol. For studying the effect of Copper ions, copper sulfate was added at a conc of 0.2, 0.3, 0.4, 0.6, 0.8 mM at different time periods after transfer to the induction medium.

### Purification and characterization of laccase

The culture filtrate of recombinant X33 cells (75 ml) was concentrated using ammonium sulfate (100% saturation). The concentrated supernatant (2 ml) was subjected to gel filtration using Sephadex75 column (30 cm x 1 cm, Pharmacia) placed in tandem with a Superdex G200 column (30 x 1 cm, Pharmacia) using AKTA FPLC system. Elution was carried out with 20 mM Tris-Cl buffer, pH 8.0 as mobile phase at a flow rate of 0.2 ml/min. Fractions of 2 ml were collected and the presence of protein monitored by measuring OD_280_. Alternate fractions of protein containing tubes were assayed for laccase activity using ABTS as the substrate [35]. The fractions showing laccase activity were pooled, dialyzed against distilled water and concentrated by lyophilization. The purity of the enzyme was checked on 15% SDS and activity was confirmed by zymogram analysis [15]. Laccase was also purified from the culture filtrate of *C. bulleri* to a specific activity of ~240 U mg^-1^ protein, as described previously [15].

For biochemical characterization, active laccase was reconstituted from the lyophilized powder by suspending in distilled water to a final activity of 100 U mL^-1^. The reconstituted enzyme was used to determine pH and temperature optimum, pH and temperature stability, tolerance to chloride ions and several organic solvents using ABTS as the substrate. The experiments were performed either in a glass cuvette or in a Microtiter plate reader (Infinite M200, Tecan) for multiple laccase measurements. Laccase activity was measured in a UV/VIS spectrophotometer (Uvikon 860) using a cuvette of 1 ml (coat thickness 1 cm) containing 50 mM Na citrate buffer (based on the observation that the pH optimum was 4.0, see below), 10 μM ABTS and appropriately diluted enzyme solution. Assay conditions were scaled down in microtitre plates (total volume 200 μl). Adequate units (~0.5-1.0 U) of laccase (aqueous solution of the lyophilized prep) were added and absorption was monitored at 420 nm. The buffers used were 0.3 M Glycine HCl buffer (pH 2 and 3), 0.3 M Na citrate buffer (pH 4–6), 0.3 M phosphate buffer (pH 7–8), 0.3 M Tris–HCl buffer (pH 9). All buffers were strong enough to buffer the corresponding pH even after addition of the acidic ABTS solution (checked via pH electrode). The dependence of activity on pH was determined for the rLac. Optimum temperature was determined at pH 4.0. Temperature stability was determined at pH 4.0 in temperatures ranging from room temperature (25°C) to 80°C. Kinetic parameters (Km and Vmax) of the nLac and the rLac were determined towards ABTS, Guaiacol and Pyrogallol. Spectrophotometric measurements of substrate oxidation by nLac and rLac were carried out in a 2 ml reaction volume containing the test substrate in 50 mM sodium citrate buffer (pH 4). All assays were carried out with equal units of laccase activity.

### Tolerance towards chloride ions and organic solvents

Tolerance to chloride ions was determined by incubating laccase solution (0.5-1.0 U) with varying concentrations of NaCl for 2 h in a total volume of 1.5 ml. Reaction vials were stored at 4°C to rule out any effect caused due to temperature. Aliquots (80 μl) were removed at regular intervals and laccase activity measured in a microtitre plate. Similarly, tolerance to organic solvents was measured by incubating laccase with 1 ml of corresponding organic solvent (at different concentrations, v/v) for 3 h. In case of acetone, ethanol, DMSO, THF, an organic solvent/water mixture of 4, 50 and 70% (v/v) was used. Because of limited solubility of MTBE in water, only 4% (v/v) solution was tested. Organic solvent tolerance was also measured in a similar manner for the purified nLac.

All enzyme activity measurements were done twice and every value was measured three times. The variation was between 5-7%.

### Peptide mass fingerprint analysis

The purified (5–10 μg) rLac and nLac were run in 5 lanes (for each protein) of 10%SDS-PAGE using a Mini-Protean Cell (Bio-rad). The proteins were stained with Coomassie blue as per standard protocols. The bands were excised out of the gel and stored in autoclaved Eppendorf tubes. The tryptic in-gel digestion and peptide finger printing was carried out using commercial service provided by Vimta Labs Ltd on a Bruker Daltonics flexAnalysis system.

### DNA sequencing

The DNA sequencing was done using MWG DNA sequencing service (Applied Biosystems 3730xl), Germany. The complete nucleotide sequence of *C. bulleri* laccase reported in this paper has been deposited in the GenBank database under the Accession No. EU195884, version 2.

## Competing interests

The authors declare that they do not have competing interests.

## Authors’ contributions

NG carried out the molecular biological studies leading to elucidation of the complete cDNA sequence and expression of the enzyme in *P. pastoris*. NB standardized the procedure for purification of the rLac and performed extensive experiments on biochemical characterization. TK and MC purified the rLac and the nLac, performed comparative analysis on solvent and chloride tolerance and analyzed the tryptic finger print data of the enzymes. MAS supervised the work in the Berlin lab. SM was the corresponding author and supervised the overall study and contributed to the manuscript organization and writing. All authors have read and approved the manuscript.

## Supplementary Material

Additional file 1 **Figure S1. **PAGE separated proteins stained with glycoprotein stain. Equal concentration (5μg) of nLac and the rLac was loaded. The gels were stained with the Pierce glycoprotein staining kit. Lane 1: molecular weight marker, Lane 2: rLac, Lane 3: nLac, Lane 4: std glycoprotein from the kit. Click here for file

Additional file 2 **Table S1. **Kinetic parameters of the purified nLac and rLac*.* All the values represent means of duplicate measurements with a sample mean deviation of lesser than 0.5%, stands for no activity. Click here for file

Additional file 3 **Table S2. **Details of the peptide fragments generated from trypsin digestion of purified nLac from *Cyathus bulleri*. Click here for file

Additional file 4 **Table S3. **Details of the peptide fragments generated from trypsin digestion of purified rLac from *P. pastoris*. Click here for file

Additional file 5 **Table S4. **Theoretical tryptic fragments. Click here for file
