# Supplementary material for: Cloning, sequence analysis, expression of Cyathus bulleri laccase in Pichia pastoris and characterization of recombinant laccase
Source: BMC Biotechnol. 2012 Oct 23;12:75. doi: 10.1186/1472-6750-12-75 (PMC3558336; doi:10.1186/1472-6750-12-75)
Supplement: Additional file 1 — Figure S1. PAGE separated proteins stained with glycoprotein stain. Equal concentration (5μg) of nLac and the rLac was loaded. The gels were stained with the Pierce glycoprotein staining kit. Lane 1: molecular weight marker, Lane 2: rLac, Lane 3: nLac, Lane 4: std glycoprotein from the kit. [file 1472-6750-12-75-S1.docx]

**Supplemen tary Fig.1 : PAGE separated protein stained with glycoprotein stain.**


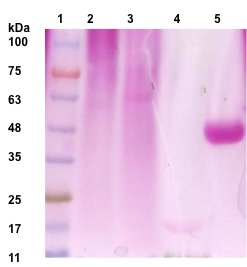


Equal concentration (5μg) of nLac and the rLac was loaded. The gels were stained with the Pierce glycoprotein staining kit. Lane 1: molecular weight marker, Lane 2: rLac, Lane 3: nLac, Lane 4:
