# Supplementary material for: Cloning, sequence analysis, expression of Cyathus bulleri laccase in Pichia pastoris and characterization of recombinant laccase
Source: BMC Biotechnol. 2012 Oct 23;12:75. doi: 10.1186/1472-6750-12-75 (PMC3558336; doi:10.1186/1472-6750-12-75)
Supplement: Additional file 2 — Table S1. Kinetic parameters of the purified nLac and rLac. All the values represent means of duplicate measurements with a sample mean deviation of lesser than 0.5%, stands for no activity. [file 1472-6750-12-75-S2.docx]

**Supplementary Table 1: Kinetic parameters of the purified nLac and rLac.**

|  |  |  | **nLac** |  | **rLac** |  |
| --- | --- | --- | --- | --- | --- | --- |
| **Substrate** | **εmax (M^-1^ cm^-1^)** | **λ max**  **(nm)** | **Km**  **( mM)** | **Vmax (U/ml)** | **Km (mM)** | **Vmax**  **(U/ml)** |
| **ABTS** | 36,000/M.cm | 420 | 0.01 | 0.02 | 0.01 | 0.02 |
| **Guaiacol** | 6,400/M.cm | 470 | 0.27 | 0.13 | 0.30 | 0.17 |
| **Pyrogallol** | 4,400/M.cm | 295 | 0.23 | 0.25 | 0.22 | 0.25 |
| **Tyrosine** |  | 280 | - | - | - | - |
|  |  |  |  |  |  |  |

All the values represent means of duplicate measurements with a sample mean deviation of lesser than 0.5%.

- stands for no activity.
