# Supplementary material for: Cloning, sequence analysis, expression of Cyathus bulleri laccase in Pichia pastoris and characterization of recombinant laccase
Source: BMC Biotechnol. 2012 Oct 23;12:75. doi: 10.1186/1472-6750-12-75 (PMC3558336; doi:10.1186/1472-6750-12-75)
Supplement: Additional file 5 — Table S4. Theoretical tryptic fragments. [file 1472-6750-12-75-S5.docx]

**Supplentary Table 4: Theoretical tryptic fragments**

| **Seq start** | **Mass (Da)** | **Peptide sequence** |  |  |  |  |  |  |
| --- | --- | --- | --- | --- | --- | --- | --- | --- |
| 3 | 424.56 | MFK |  |  |  |  |  |  |
| 29 | 2863.47 | TLYIFTLLSIAYGAIGPVLDMHIVNK | | |  |  |  |  |
| 38 | 1004.11 | VISPDGFNR |  |  |  |  |  |  |
| 61 | 2172.38 | SAVLAGGTADNADFPGPLVTGNK | | |  |  |  |  |
| 80 | 2176.45 | GDHFQLNVIDSLTDTTMLR | |  |  |  |  |  |
| 142 | 6919.57 | GTSIHWHGLFQHGTTWADGPVGVNQCPISPGNSFLYDFSVPDQAGTFWYHSHHSTQYCDGLR | | | | | | |
| 155 | 1450.61 | GPLVVYDPNDPHK | |  |  |  |  |  |
| 194 | 4146.61 | SLYDVDDESTVITLADWYHTPAPSAGLVPTTDAVLINGK | | | |  |  |  |
| 196 | 231.25 | GR |  |  |  |  |  |  |
| 215 | 1913.22 | FPTGPTSPLSVINVTPGTK | |  |  |  |  |  |
| 217 | 337.38 | YR |  |  |  |  |  |  |
| 219 | 321.38 | FR |  |  |  |  |  |  |
| 263 | 4780.42 | LVSISCDPNFVFSIDGHTFTIIEVDGVNVTPVEVDSIQIFAGQR | | | | |  |  |
| 280 | 2099.33 | YSFVLNANQPVDNYWIR | |  |  |  |  |  |
| 287 | 740.89 | AKPNIAK |  |  |  |  |  |  |
| 301 | 1419.6 | GVTFDGGINSAILR | |  |  |  |  |  |
| 356 | 5530.09 | YAGAPDTDPTTSQTPNSAPMVETDLHPLENPGAPGGSNPADVPLNLAIAFGSNLK | | | | | |  |
| 398 | 4326 | FTVNGATFAPPNVPVLLQILSGAQTAQDLLPTGSVYTLPANK | | | | |  |  |
| 426 | 3068.51 | VIEISIPGGTTGFPHPFHLHGHTFDVVR | | |  |  |  |  |
| 440 | 1528.598 | SAGSSVYNYDNPVR | |  |  |  |  |  |
| 441 | 174.2 | R |  |  |  |  |  |  |
| 456 | 1459.53 | DAVNTGGAGDNVTIR | |  |  |  |  |  |
| 513 | 6201.02 | FLTDNAGPWILHCHIDWHLELGLAIVFAEDVPTIAASNPPDAWDNLCPAYATQPTGT | | | | | |  |

| yellow | Detected unglycosylated Peptide fragments. |
| --- | --- |
| Pink | Detected glycosylated fragment. |
